# Direct and Indirect Effects of Ivermectin on Phytophagous, Frugivorous and Parasitoid Insects

**DOI:** 10.3390/insects16040366

**Published:** 2025-04-01

**Authors:** Lars Laber, Nico Blüthgen, Karsten Mody

**Affiliations:** 1 Department of Entomology, University of California—Riverside, Riverside, CA 92521, USA; 2 Ecological Networks, Department of Biology, Technical University of Darmstadt, 64287 Darmstadt, Germany; bluethgen@bio.tu-darmstadt.de; 3 Department of Applied Ecology, Hochschule Geisenheim University, 65366 Geisenheim, Germany

**Keywords:** ivermectin, sublethal effects, trophic stages, feeding experiments, herbivores, parasitoids

## Abstract

Ivermectin, an anthelmintic used in livestock, is excreted in faeces and can therefore affect non-target organisms. While its effects on coprophagous insects have been well studied, recent research suggests that it can be taken up by plants, raising the possibility that it may affect a much wider range of invertebrates than previously known. Our study investigated the effects of ivermectin on insects from different feeding guilds. First, we tested its effects on the larvae of the noctuid moths *Spodoptera frugiperda* and *Helicoverpa armigera* (chewing herbivores) using an artificial diet. Secondly, we investigated the effects of its presence in the soil of pea plants *Pisum sativum* on the pea-feeding aphid *Acyrthosiphon pisum* (phloem-feeding herbivore). Thirdly, we tested ivermectin in an artificial diet for *Drosophila melanogaster* used as a host for the parasitoid *Pachycrepoideus vindemmiae*. Our results showed that ivermectin reduced pupal weight and survival in moth larvae, inhibited aphid colony establishment and reduced parasitoid emergence rates from ivermectin-exposed *Drosophila* pupae. These results highlight the potential of ivermectin to affect multiple trophic levels and emphasise the need for sustainable veterinary practices in the use of anthelmintics in free-ranging livestock and companion animals.

## Simple Summary

Our study evaluated the effects of ivermectin in sublethal doses on insects of different feeding guilds. We investigated the effects of ivermectin on weight and development when applied to the diet of two chewing herbivores, *Spodoptera frugiperda* and *Helicoverpa armigera*; the success of colony establishment of *Acrythosiphon pisum* on *Pisum sativum* grown in ivermectin-laced soil; and the reproductive success of the parasitoid *Pachycrepoideus vindemmiae* when parasitizing *Drosophila melanogaster* larvae fed with an ivermectin-laced diet. We found that in all experiments conducted in this study, ivermectin had significant negative effects on the insects involved, such as a reduction in pupal weight, prolonged development time, less successful establishment and reduced emergence from pupae. Since ivermectin and other macrocyclic lactones with similar properties are widely used as anthelmintics—dewormers for mammals—they are excreted into the environment in large amounts and can be taken up in similar circumstances such as those we have investigated here. We conclude that ivermectin can affect insects of different feeding guilds and have significant effects on different ecosystem processes. We recommend that ivermectin and comparable anthelmintics be used more cautiously in accordance with their negative effects on the environment and that the sublethal effects of seemingly harmless substances be researched more intensively in general.

## 1. Introduction

Macrocyclic lactone (ML) anthelmintics are frequently used in farming, where they are used to deworm livestock such as cattle and sheep [1]. Ivermectin (IVM) is one of the most frequently used MLs [2]. It is a mixture of two metabolic products of the bacterium *Streptomyces avermectinius* [2]. In invertebrates, IVM blocks neurotransmissions by blocking specific glutamate-controlled chloride channels found in nematodes and arthropods. This leads to paralysis of pharyngeal and somatic muscles and thus ultimately to the invertebrates’ death [2]. Mammals and other vertebrates are not affected by these effects, as their chloride channels are structured differently [2,3,4]. IVM can be administered to the animal as an injection, as a bolus, orally or as a pour-on via direct skin contact [5].

MLs are excreted into the environment in large quantities because they are only weakly metabolised in the treated animals [6,7]. Since they are also highly lipophilic, they tend to adhere to the dung [6]. This poses a great risk for non-target organisms, especially coprophagous insects such as dung beetles or dung flies, which are either poisoned themselves or put their offspring in great risk when they have to feed on contaminated dung [8,9,10]. IVM residues can still be detected several weeks after treatment in dung and the half-life of these substances varies greatly depending on outside conditions, so dung heaps can stay poisonous for weeks or even months after excretion, being potentially harmful and hindering degradation [11,12]. Also, ML molecules can be washed out with smaller pieces of dung via rain, seep into the soil or groundwater or be trampled into the soil, where it could then affect not only seed banks but also subterranean and aquatic species, where severe effects have been reported [13,14,15]. In addition to affecting the fauna, multiple experiments have reported that an uptake of MLs, especially IVM, into plants is possible both in the lab and also on the field [16,17,18]. IVM and other MLs were reported to lead to significant changes in growth and metabolism in plant structures [17,19], even affecting seed germination and the emergence of seedlings [20,21,22].

Several studies have shown that IVM can be taken up by plants and affect them negatively [16,23,24]. Laboratory experiments have shown the effects of IVM on multiple plant species, such as *Glycine max*, *Plantago lanceolata* and *Sinapis alba* [16,17,23,24]. Effects can range from the expression of stress hormones to shortened root growth. Molecules were identified in the roots, stems and leaves. Since these molecules must have been transported through the stem, to the leaves, it can be assumed that they travel within the xylem sap, which makes them accessible to both chewing and sucking herbivores. While the effects of macrocyclic lactones and especially IVM in coprophagous insects are well studied [8,9,25], the effects on other groups of insects, such as chewing and sucking herbivores, have not been studied in detail yet.

Besides its direct uptake from plant material, the indirect uptake of IVM by carnivores (predators or parasitoids) feeding on chewing or sucking herbivores must also be considered. While species have shown varying levels of tolerance to IVM and other macrocyclic lactones, larval stages, in general, are the most vulnerable to various kinds of toxins. Therefore, parasitoids and especially their offspring would be affected if their host had ingested IVM prior to parasitisation. This suggests that IVM can affect plants and animals on multiple trophic levels, influencing whole ecosystems. Our goal was to broaden the perspective on effects of this important and widespread veterinary medicine beyond case studies on lethal doses on few non-target organisms. Correspondingly, a recent study on sublethal concentrations of agrochemicals described various unexpected effects for over 1000 substances (not including ivermectin) and highlighted the potential for the additive impacts of cocktails of such substances, as well as increasing temperature [26].

In our study, we conducted experiments on a variety of insects with different feeding habits. For the direct effects of ivermectin on chewing herbivores, we fed *Spodoptera frugiperda* and *Helicoverpa armigera*, two lepidopteran species, with an artificial diet spiked with ivermectin. *Helicoverpa armigera* (Noctuidae) is one of the largest agricultural pests in Australia, China and India [27]. As *H. armigera* is a migratory species and often infests crops, it has been introduced all over the world but is usually found in warmer areas around the equator [27,28]. *Spodoptera frugiperda* (Noctuidae) originates from tropical and subtropical regions of the Americas, but it has been introduced to Africa and India, where it causes severe damage to important agricultural crops such as corn and alfalfa [29].

To test the impact of ivermectin on sucking herbivores when taken up by plants from the soil, we reared *Pisum sativum* in ivermectin-spiked soil and placed a single *Acyrthosiphon pisum* on *P. sativum*. *Pisum sativum* is a temperate legume common in Europe, where it is cultivated to feed humans and animals [30,31]. *Acyrthosiphon pisum* is a sucking pest which causes major losses in *P. sativum* crops, causing *P. sativum* to wither when too many individuals suck from its phloem [32].

To check the impact of ivermectin on parasitoids when ingested by the hosts, we reared *D. melanogaster* to the pupal stage on a diet spiked with ivermectin and let the parasitoid *Pachycrepoideus vindemmiae* parasitize them.

The generalist pupal parasitoid *Pachycrepoideus vindemmiae* has a wide host spectrum in Diptera and also hyperparasitizes beneficial Hymenoptera [33]. *Pachycrepoideus vindemmiae* is solitary and is one of two species effectively used in Europe and the USA to control the cherry vinegar fly (*Drosophila suzukii*) [34]. Female *P. vindemmiae* lay up to 40 eggs per day between the pupal body and puparium of their hosts. Before the eggs are laid, the pupa is permanently paralysed by the toxin of *P. vindemmiae*, which also stops the development of the host animal. *Pachycrepoideus vindemmiae* needs about three weeks (20.8 days in females and 20.1 days in males) at about 23 °C for complete development after laying until hatching from the pupa [34]. *Pachycrepoideus vindemmiae* also does host-feeding, which leads to an improvement in the quality and quantity of the laid eggs. This has no negative effects on the larvae of *P. vindemmiae* that live on the infected pupae [33]. *Drosophila melanogaster* (Drosophilidae) is a common model organism in biological research. It has a short generation time and a high fecundity, making it easy to rear in large quantities. The larvae feed on rotten fruit, yeast and other fermenting substances [35].

In our study, three experiments were conducted to answer the following questions about the direct and indirect effects of IVM on insects of different trophic levels:

- Does the uptake of IVM directly affect the growth and development of chewing herbivores?
- Does IVM in soil indirectly affect the establishment and growth of aphid colonies on plants growing in IVM-treated soil?
- Does the uptake of sublethal doses of IVM by host insects indirectly affect the development of parasitoids feeding on these hosts?

## 2. Materials and Methods

### 2.1. Ivermectin

Ivermectin (IVM) was obtained as a pure substance (purity ≤ 95%) from Alfa Aesar (now ThermoFisher, Landau, Germany) for all experiments. IVM was first dissolved in ethanol 96% (denatured, unless otherwise described) to allow for the most homogeneous distribution possible.

### 2.2. Direct Effects of Ivermectin on Lepidopteran Larvae

#### 2.2.1. Test Species

We received eggs of both *Spodoptera frugiperda* and *Helicoverpa armigera* on request from Bayer CropScience (Monheim, Germany).

#### 2.2.2. Artificial Diet

We reared noctuid larvae with an artificial diet for noctuids according to a recipe from Bayer Crop Science (Bayer CropScience, Monheim, Germany; [36]). The artificial diet was prepared in advance, frozen and used as required.

#### 2.2.3. Feeding Experiment with L3-L4 *Spodoptera frugiperda*

IVM was dissolved in 96% ethanol and added to the diet in 100 µL ethanol doses while still in liquid form and stirred with a magnetic stirrer. We produced a control diet with ethanol but without IVM and additional diets with concentrations of 200, 20, 5, 1.5 and 0.3 µg IVM per g diet (fresh weight). After hatching, the caterpillars were reared on artificial diet without IVM as a group. Caterpillars were kept at a constant temperature of 26 °C, a humidity of 60% and a day/night cycle of 16/8 h throughout the experiment.

After reaching the 4th–5th larval stage, 20 caterpillars for both the IVM and control groups were isolated individually in Petri dishes, with the corresponding diets containing different concentrations of IVM and a control group without the anthelmintic. Petri dishes were randomly placed in the climate cabinets. The Petri dishes contained filter paper to absorb excess moisture and faeces. The dishes were moistened regularly to prevent dehydration. Food was provided ad libitum. The caterpillars were weighed before starting the experiment and after pupation. The experiment was concluded after 12 days, when every caterpillar had pupated or died.

#### 2.2.4. Feeding Experiment with L1 *Spodoptera frugiperda* and *Helicoverpa armigera*

IVM was dissolved in 96% ethanol and added to the artificial diet before it had cooled down and solidified completely. We produced concentrations of 200, 20, 5, 1.5 and 0.3 µg IVM per g diet. While still in liquid form, the diet was stirred with a magnetic stirrer for at least 15 min to ensure homogenous distribution of the ethanol solution. We produced a control diet with the same amount of ethanol but without IVM. After hatching, 10 larvae per species for each IVM concentration and the control group were immediately isolated in individual Petri dishes containing the corresponding diet. The caterpillars were kept at a constant temperature of 26 °C, humidity of 60% and a day/night cycle of 16/8 h throughout the experiment.

The Petri dishes contained filtration paper to absorb excess moisture and faeces. The dishes were moistened regularly to prevent dehydration. Food was provided ad libitum. The status of the caterpillars was checked two times per week; dead individuals were removed, and the day of death was recorded. Weight and sex were noted after pupation. The experiment was concluded after 27 days when all remaining individuals had pupated.

### 2.3. Host Plant-Mediated Effects of Ivermectin on the Phloem-Feeding Herbivore Acyrthosiphon pisum

We used the common garden crop pea *Pisum sativum* (Fabaceae), variety “Kleine Rheinländerin” (ZG Raiffeisen, Karlsruhe, Germany), and its common antagonist, the pea aphid *Acyrthosiphon pisum* (Aphididae).

Peat substrate tabs were purchased from Jiffy Products International BV (Zwijndrecht, The Netherlands). These were infused with 80 mL tap water. We prepared 25 pots for each concentration used in our experiment (20 µg g^−1^, 1.5 µg g^−1^ and 0 µg g^−1^ IVM per g of soil (wet weight at start of experiment)), 75 pots in total. IVM was dissolved in ethanol to prepare stock solutions for our test concentrations and 1 mL of the appropriate IVM/ethanol solution was added to each pot. Control pots were treated only with 1 mL ethanol. After the solution was mixed with the substrate, pots were placed in a fume hood for 24 h for the ethanol to dissipate. Afterwards, three seeds of *P. sativum* were added to each pot. Then, the pots were placed in a climate cabinet with a day/night cycle of 16/8 h at 23/17 °C and a humidity of 70% for 26 days before aphids were placed on the plants.

We cultivated several additional *P. sativum* plants under the same conditions, but without ethanol and IVM in the substrate as plants to rear our test subjects on. *Acyrthosiphon pisum* were ordered from MD-Terraristik (Kleinostheim, Germany). After purchase, the aphids were transferred from the breeding cups to rearing plants and kept there until the experiment began (November 11). The aphids were kept in climate chambers with a day/night cycle of 14/10 h and a temperature of 20/17 °C during the whole experiment. At the start of the experiment, one newborn *A. pisum* nymph was placed on each *P. sativum* plant.

Aphids were examined four days after the start of the experiment and counted weekly thereafter. The pea plants were watered twice a week. The experiment was terminated after 25 days, when the first plants started to break down. Plants that died during the experiment were not included in the calculations.

### 2.4. Host-Mediated Effects of Ivermectin on the Parasitoid Pachycrepoideus vindemmiae

We obtained *P. vindemmiae* specimens from Dr. Anette Herz at the Julius-Kühn-Institute Dossenheim on request. Before the start of the experiment, parasitoids were sucrose-fed and had not previously been exposed to fly pupae.

*D. melanogaster* specimens were kept on apple–banana pulp of the brand “REWE Bio” (REWE, Cologne, Germany) during the whole trial period. For this purpose, specimens were kept in breeding vessels on untreated apple–banana pulp and transferred to other vessels with treated pulp for experiments.

#### Parasitisation Experiment

IVM was dissolved in 100 µL pure ethanol (99%) to create a concentration of 0.03 µg IVM per g pulp. The apple–banana pulp was stirred by a magnetic stirrer, the IVM–ethanol solution was added and the stirring continued for 15 min. The pulp was then divided into ten vials, which were then placed in a fume hood for one day to allow the ethanol to evaporate as much as possible. For the control group, the same procedure was used, except no IVM was added to the ethanol. *D. melanogaster* were added to the vials from pre-existing cultures and were removed two days later after laying eggs. The vials were kept in a climate cabinet at a constant temperature of 25 °C, humidity of 70% and a day/night cycle of 16/8 h until several larvae had started to pupate. The pupae were removed from the vials, cleaned carefully with water, counted and transferred to new vials. Two *P. vindemmiae* females, fertilised before the start of the experiment, were placed in each vial and removed after two days. During and after parasitisation, the pupae were kept at 23 °C, 70% humidity and a day/night cycle of 16/8 h. The average development time of *P. vindemmiae* is 23 days; therefore, the vials were observed until the 31st day after contact with *P. vindemmiae*. Hatched individuals were counted, captured and frozen.

### 2.5. Data Analysis

Before ANOVA, all data were visually inspected to check whether the assumptions of the applied inferential statistics were met. Statistical analysis was performed with Statistica™ 13.3 (StatSoft Inc., Tulsa, OK, USA) and graphs were created using ORIGIN^®^ (ORIGINLab Corporation, Northampton, MA, USA). All data are available in Supplementary File S1.

## 3. Results

### 3.1. Effects of Ivermectin Concentration on Larval Performance (Development Time and Pupal Mass) of a Chewing Herbivore

**Figure 1:**
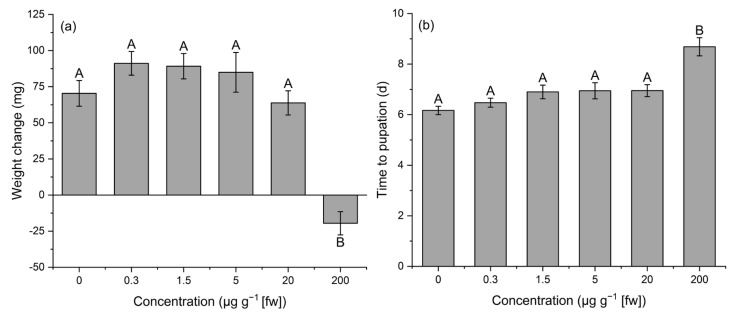
Effects of different ivermectin concentrations on weight change from start of the experiment to pupation (**a**) and days until pupation (**b**). Different letters indicate significant differences (*p* ˂ 0.05 Tukey HSD Test).

We found that IVM concentration had a significant effect on the weight change and days to pupation of *S. frugiperda* larvae, while larval sex and the interaction of IVM concentration and sex had no effect (Table 1). The highest IVM concentration (IVM; 200 µg g^−1^) in the diet had a significant inhibitory effect on the development of *S. frugiperda*, as evidenced by a loss of 19.53 ± 7.81 mg in mean weight from the beginning of the experiment to pupation, compared to an increase in mean weight by 70.39 ± 11.29 mg in the control group (0 µg g^−1^ IVM) (Figure 1a). The development time was also influenced, with the concentration of 200 µg g^−1^ IVM leading to a significant increase in the time to pupation of 2.5 days compared to the control group (Figure 1b). No other concentrations were significantly different from the control.

### 3.2. Effects of Ivermectin Concentration on Larval Mortality of Two Chewing Herbivores

**Figure 2:**
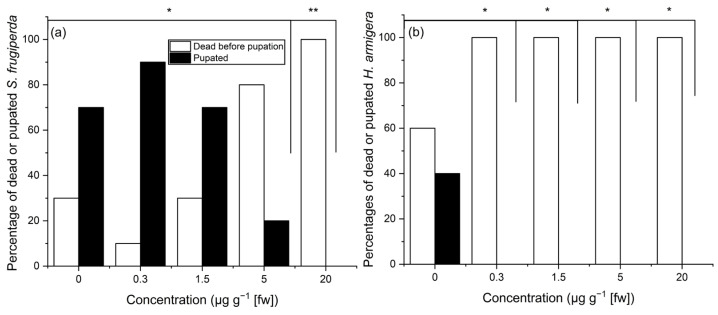
Percentage of dead or pupated *Spodoptera frugiperda* (**a**) and *Helicoverpa armigera* (**b**) until pupation when feeding on artificial diet containing different concentrations of ivermectin. Brackets indicate significant differences between control (0 µg g^−1^ IVM) and the respective column according to Fisher’s exact probability test (one-sided expectation; * *p* < 0.05, ** *p* < 0.01).

The effects of IVM on the larvae of two lepidopterans of the same family, Noctuidae, varied substantially. *S. frugiperda* showed a higher percentage of pupated than dead individuals at 0 (70%), 0.3 (90%) and 1.5 (70%) µg g^−1^ IVM, while *H. armigera* did not survive on any diet containing IVM but also showed a low baseline survival at 0 (40%) µg g^−1^ IVM (Figure 2). The number of pupated individuals of *S. frugiperda* differed significantly from the control group at 5 (20%; *p* = 0.03, Fisher’s exact probability test, one-sided expectation) and 20 (0%, *p* = 0.003) µg g^−1^ IVM, while in *H. armigera*, the percentage of pupated individuals was significantly lower (0) than in the control group (40%, *p* = 0.04) at all IVM concentrations.

### 3.3. Host Plant-Mediated Effects of Ivermectin Concentration on Colony Establishment of a Sucking Herbivore

**Figure 3:**
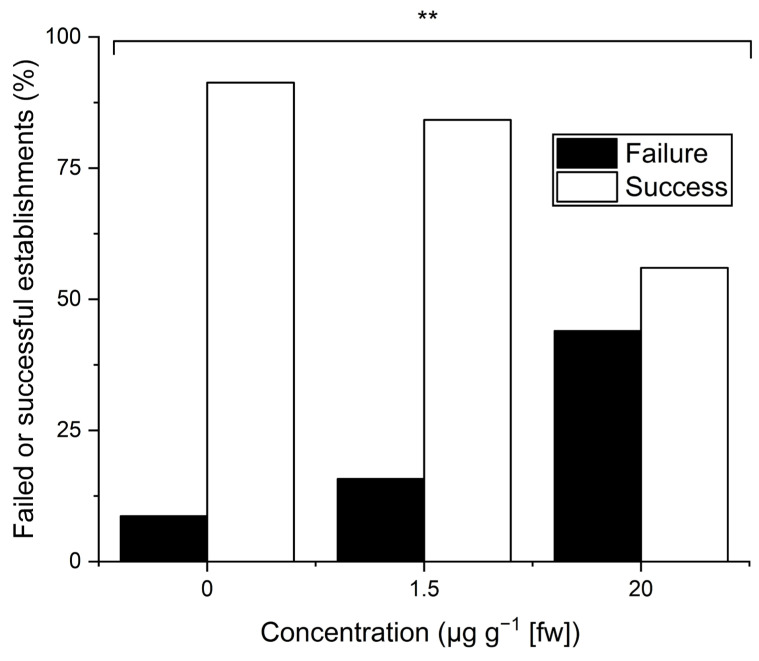
Colony establishment of pea aphids on pea plants treated with differing concentrations of ivermectin (IVM). Brackets indicate significant differences between control and respective columns according to Fisher’s exact probability test (two-sided expectation; ** *p* < 0.01).

Aphid colony establishment on pea plants growing in substrate with a concentration of 20 µg g^−1^ IVM was significantly lower (14 (56%) successful, 11 (44%) failed, Figure 3) than colony establishment on plants growing in soil with no added IVM in substrate (21 (91.3%) successful, 2 (8.7%) failed; *p* = 0.008, Fisher’s exact probability test, two-sided expectations). Between the untreated substrate and the substrate with 1.5 µg g^−1^ IVM (16 (84.2%) successful, 3 (15.8%) failed), no significant differences in colony establishment were found (*p* = 0.64), while between the substrate with 20 µg g^−1^ IVM and 1.5 µg g^−1^ IVM, a seemingly strong but non-significant trend was found (*p* = 0.058).

### 3.4. Host-Mediated Effects of Ivermectin on Parasitoid Development

**Figure 4:**
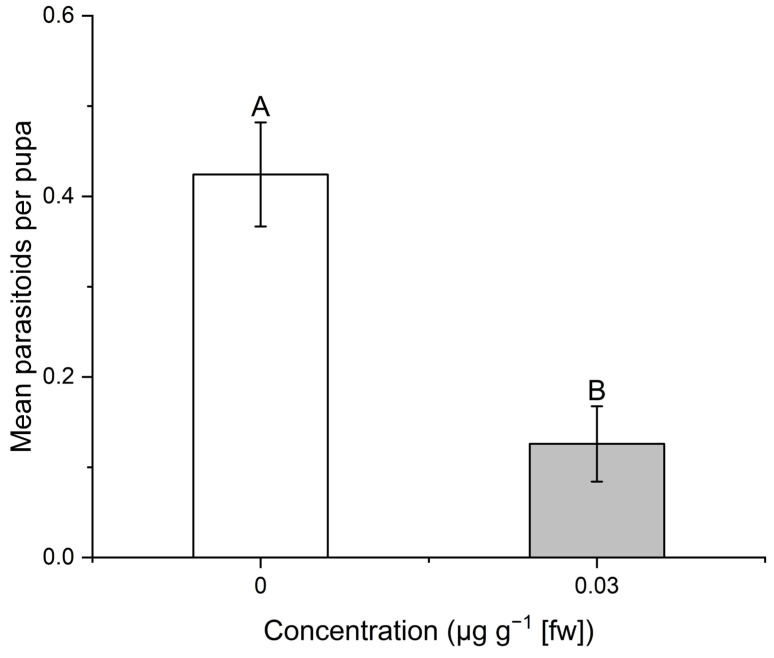
Mean number of *Pachycrepoideus vindemmiae* parasitoids per pupa reared on ivermectin spiked diet (0.03 µg g^−1^) or control diet. n = 194 control pupae, 137 IVM-treated pupae, divided into 10 vials for each treatment. Different letters indicate significant differences (*p* ˂ 0.05, *t*-test).

Pupae of *D. melanogaster* that had consumed IVM during larval development produced significantly fewer *P. vindemmiae* parasitoids (0.13 parasitoids/pupa) than pupae that developed without IVM (0.42 parasitoids/pupa) (t (18) = 4.2, *p* ≤ 0.001; Figure 4).

## 4. Discussion

### 4.1. Improving the Understanding of the Environmental Impact of Ivermectin

More recently, studies have begun to shed light on the herbicidal effects of IVM, revealing adverse effects on plants [17,19]. These observations suggest that the environmental reach of IVM is broader than originally understood, affecting multiple facets of ecosystem dynamics.

Our study advances this field of research by providing new insights into the effects of IVM on organisms at previously unexplored trophic levels. It thereby highlights the potential for IVM residues to affect a wider range of biotic interactions and ecosystem processes than previously recognised. By filling the gaps in our understanding of the ecological footprint of IVM, our study contributes knowledge that can inform environmental risk assessments and guide the development of more sustainable practices for veterinary drug use.

### 4.2. Direct Diet-Mediated Effects of IVM on Lepidoptera Larvae

We found that IVM, when applied in a later stage of Lepidoptera larval development, can effectively reduce weight gain and extend development time until pupation in the chewing herbivore *Spodoptera frugiperda*. These results thus demonstrate that IVM can have a significant negative impact on late instar larvae of chewing herbivores when administered in sublethal doses [26]. The larvae that were fed the diet with the highest concentration of IVM reached the pupation stage but were unable to develop a complete chrysalis. This may have resulted in the desiccation and mortality of the larvae. It is known that IVM inhibits chitin production, which may explain the reduction in larval growth and the incomplete formation of the chrysalis [37,38]. The results, which demonstrated that larvae exposed to lower doses of IVM survived, pupated and matured into adults, suggest that at least late instar larvae of *S. frugiperda* may have the ability to metabolise or excrete low concentrations of IVM, thereby reducing the harmful effects of IVM.

When Lepidoptera larvae were exposed to IVM throughout their larval development, strong differences in tolerance to IVM were observed even between the closely related noctuid species studied. This finding emphasises that the effects of IVM on non-target organisms can vary widely and that the reactions of the more sensitive species in particular must be carefully considered to avoid strong negative effects of IVM application. This is particularly relevant when considering that IVM would most likely enter the environment on pastures, where it would mainly affect grassland plant species and the herbivores that feed on them [21,22]. These could include generalists and rare specialists with important roles in the ecosystem.

### 4.3. Indirect Plant-Mediated Effects of IVM on Aphids

Looking at the establishment of aphid colonies on pea plants to investigate the effects of IVM on non-target herbivores, our results showed that IVM can also have a significant negative effect on sucking herbivores. In our experiments, this was particularly the case when the soil was treated with a high concentration of IVM (20 µg g^−1^).

The simplest explanation for these observations is that IVM was taken up by the plants and subsequently affected the aphids feeding on the plant, resulting in poor aphid performance. Alternatively, IVM uptake may have influenced the plants themselves. Previous studies have shown that macrocyclic lactones, including IVM, can be taken up by plants and alter their growth and metabolic processes [16,17,23]. Such changes could weaken the plants and potentially reduce the availability or quality of nutrients transported in the vascular tissue of the plants, which in turn creates less favourable conditions for aphids that rely on these nutrients.

While previous research has documented the effects of IVM on root growth [17], we found no visible differences between the groups of plants we used. This suggests that even without overt changes in plant morphology, subtle physiological or metabolic alterations induced by IVM may still negatively affect herbivores. These findings therefore suggest a novel plant-mediated pathway by which IVM can impact sucking herbivores and emphasise the need to consider indirect effects when assessing the environmental risks of veterinary drugs.

At a lower IVM concentration, there were no significant differences in aphid population size and growth compared to the control group. There could be several reasons for this. Since lower concentrations had no significant effect on colony establishment, it could be assumed that not enough IVM reached the plant, as IVM binds strongly to the soil [39] and the outside of the roots [17,23]. A low concentration may not even reach the inside of the plant. A higher concentration allows more molecules to enter the plant. In addition, IVM is metabolised in the soil by natural degradation and bacteria [6,40] and in plants [16], which also reduces the concentration in the plant itself. Only at higher concentrations could IVM reach the stem and leaves where it could be taken up by the aphids and affect them. The finding that we did not observe the same response in all plants treated with the highest IVM concentration may indicate that the amount of IVM in the plants varied due to differences in plant growth, especially root growth. Thus, more or less of the IVM distributed in the soil could have been taken up by the plants. Furthermore, plants may have different abilities to metabolise IVM at the individual level. This has been shown in previous studies for plants of the same family, which differed in their response to moxidectin, a macrocyclic lactone comparable to ivermectin [22], resulting in differing concentrations of moxidectin in the xylem.

### 4.4. Indirect Host-Mediated Effects of IVM on Hymenopteran Parasitoids

In addition to the negative effects of IVM on different species of insect herbivores, our results suggest that IVM exposure may also indirectly affect higher trophic levels, as shown by the significantly reduced hatching of hymenopteran parasitoids from host pupae. The residues of IVM in *Drosophila melanogaster* pupae may have been sufficient to paralyse or kill the larvae of the parasitoid *Pachycrepoideus vindemmiae*. These larvae are very small and therefore likely to be susceptible to very low doses of IVM that would not have been high enough to kill the host pupae. If this interpretation is correct, IVM could even be considered to protect herbivores against parasitoids when ingested by herbivores at sublethal doses [26]. This greater susceptibility of parasitoids to IVM compared to their host herbivores can have several important consequences, including disruption of natural pest control, ecosystem imbalances and also economic consequences. Understanding and managing the different sensitivities of parasitoids and herbivores to IVM and other pesticides is therefore crucial for sustainable pest control and the maintenance of functioning ecosystems.

## 5. Conclusions

The results of our study show that IVM has remarkable direct and indirect effects on various non-target insect species belonging to different trophic levels. This means that IVM, like other pesticides [41], can have different negative effects on non-target organisms. While previous research has demonstrated that IVM can be taken up by plants and distributed to different tissues [16,17,23], our study has shown that the effects of IVM can reach further and harm insects when they feed on these IVM-containing plant tissues. Furthermore, these indirect IVM effects not only impact herbivores that feed on IVM-containing plants, but can also affect natural enemies of herbivores, including beneficial parasitoids that feed on IVM-contaminated herbivores. These findings on direct and indirect effects of IVM on non-target organisms belonging to different trophic levels emphasise the urgent need for additional studies to identify the pathways by which insects and other invertebrates come into contact with macrocyclic lactones, including IVM. A comprehensive understanding of how macrocyclic lactones affect organisms at different trophic levels is essential to manage their sustainable use and mitigate their adverse effects. This includes the potential loss of biodiversity and the degradation of ecosystems due to the release of IVMs by free-ranging livestock and pets treated with IVM.

## Figures and Tables

**Table 1:** Results of ANOVA on the effects of ivermectin concentration (IVM) and sex (S) on weight change and days to pupation; N = 115; 5 out of 120 died before pupation.

		Change in Weight			Days to Pupation		
	DF	MQ	F	*p*	MQ	F	*p*
**Ivermectin concentration (IVM)**	**5**	**29,794.3**	**17.8**	**≤0.0001**	**14.1**	**10.7**	**≤0.0001**
**Sex (S)**	1	201.8	0.1	0.73	0.5	0.4	0.52
**IVM × S**	5	2353.3	1.4	0.23	1.7	1.3	0.26
**Error**	102	1674.2			1.3		

## Data Availability

The original contributions presented in this study are included in the article. Further inquiries can be directed to the corresponding authors.

## Supplementary Materials

The following supporting information can be downloaded at https://www.mdpi.com/article/10.3390/insects16040366/s1, Supplementary File S1. “Dataset”.

## Abbreviation

The following abbreviations are used in this manuscript:

IVM	Ivermectin
